# Actor-recipient role affects neural responses to self in emotional situations

**DOI:** 10.3389/fnbeh.2015.00083

**Published:** 2015-04-15

**Authors:** Xiaoyan Wang, Li Zheng, Xuemei Cheng, Lin Li, Lining Sun, Qianfeng Wang, Xiuyan Guo

**Affiliations:** ^1^School of Psychology and Cognitive Science, East China Normal UniversityShanghai, SH, China; ^2^Key Laboratory of Brain Functional Genomics, Ministry of Education, Shanghai Key Laboratory of Brain Functional Genomics, East China Normal UniversityShanghai, SH, China; ^3^Shanghai Key Laboratory of Magnetic Resonance, East China Normal UniversityShanghai, SH, China; ^4^Shanghai Key Laboratory of Magnetic Resonance and School of Psychology and Cognitive Science, East China Normal UniversityShanghai, SH, China

**Keywords:** self-evaluation, actor-recipient role, ACC, dmPFC, OFC

## Abstract

People often take either the role of an actor or that of recipient in positive and negative interpersonal events when they interact with others. The present study investigated how the actor-recipient role affected the neural responses to self in emotional situations. Twenty-five participants were scanned while they were presented with positive and negative interpersonal events and were asked to rate the degree to which the actor/the recipient was that kind of person who caused the interpersonal event. Half of the trials were self-relevant events and the other half were other-relevant events. Results showed that people were more likely to isolate self from negative events when they played the role of actor relative to recipient. Pregenual anterior cingulate cortex (pgACC) and posterior dorsal anterior cingulate cortex (pdACC) were more active for self than other only in negative events. More importantly, also in negative interpersonal events, dorsal medial prefrontal cortex (dmPFC) showed greater self-related activations (self-other) when participants played the role of recipient relative to actor, while activities in orbitofrontal cortex (OFC) were greater for self than other only when the evaluation target played the role of recipient. These results showed that the actor-recipient role affected neural responses to self in emotional situations, especially when a recipient role was played in negative situations.

## Introduction

“Self” is a unique mental construct and self-processing is functionally dissociable from other forms of processing within the human brain (Kelley et al., 2002). Neuroimaging studies have demonstrated the involvement of the cortical midline structures (CMS), including medial prefrontal cortex (mPFC), posterior cingulate cortex (PCC) and precuneus, in self-processing (Kelley et al., 2002; Beer and Hughes, 2010; Korn et al., 2012). In these studies, participants were asked to rate self-descriptiveness and other-person-descriptiveness of favorable and unfavorable trait words. Meta-analytic evidence has suggested a spatial gradient within mPFC, i.e., ventral mPFC (vmPFC) and dorsal mPFC (dmPFC), based on the distinct functions during self-processing (Denny et al., 2012). Specifically, vmPFC is engaged in encoding self-relevance of stimuli, whereas dmPFC is involved in self-evaluation and self-related reappraisal (Northoff and Bermpohl, 2004; Beer and Hughes, 2010; Han et al., 2010; Korn et al., 2012).

One typical character of self-processing is that it often interacts with emotional valence (Moran et al., 2006). When considering personal relevance of information, individuals are more likely to endorse positive information as self-descriptive and isolate negative information (Mezulis et al., 2004; Moran et al., 2006; Beer and Hughes, 2010; D’Argembeau et al., 2012). Neuroimaging studies have demonstrated that brain activities in anterior cingulate cortex (ACC) and orbitofrontal cortex (OFC) were associated with interactions between self-processing and emotional valence (Moran et al., 2006; Sharot et al., 2007; Beer and Hughes, 2010; Seidel et al., 2010; Korn et al., 2012). Specifically, ACC was associated with conflict monitoring (Carter et al., 1998; Bush et al., 2000; Northoff and Bermpohl, 2004; Etkin et al., 2011), while OFC was associated with accurate self-view (Beer et al., 2006, 2010; Beer, 2007; Beer and Hughes, 2010). In addition, previous studies had demonstrated that people were especially sensitive to self-related negative information relative to positive information. They would preferentially process negative information, indicating “negativity bias” (Carretié et al., 2001; Huang and Luo, 2006; Hilgard et al., 2014). Neuroimaging studies have proposed that ACC and mPFC played a critical role in negative emotional processing (Ochsner et al., 2004; Cheng et al., 2010; Etkin et al., 2011; Guo et al., 2012).

Self-processing is not only to judge self-descriptiveness of trait words, we also, at various moments in life, might have cause to evaluate ourselves in interpersonal situations. No self-other difference in interpersonal situations is better known than the actor–observer asymmetry in social and cognitive psychology (Jones and Nisbett, 1971; Malle, 2006). Remarkably, when self plays the role of an actor (actively performs the action), he/she tends to attribute negative events to situations and positive events to dispositions of themselves, while a reverse attribution tendency is found when she/he is just observing the other-relevant event as a passive observer (Malle, 2006). Since the actor is referred to self actively performing the action in the self-relevant event and the observer condition involved the self-irrelevant events, it has been widely acknowledged that the classic actor–observer asymmetry was due to the involvement of self-processing (Cunningham et al., 1979; Malle, 2006). However, in social interpersonal situations, self also often plays the role of a recipient who actively observes the action from the actor in self-relevant events.

Although interpersonal situations, in which self played either the actor or recipient role, seem to be self-related, previous studies have argued that there were decreased self-other attribution differences for the recipient relative to actor (Kasof and Lee, 1993; Malle, 2006). For example, Kasof and Lee (1993) showed that, compared to accurate attributions in other-relevant events, individuals tended to attribute more positive (relative to negative) events to self when they played the role of actor, while this self-serving evaluations decreased when they played the role of recipient. Previous research had claimed that self-serving evaluations were heuristic judgments (Dunning et al., 1989; Chambers and Windschitl, 2004; Beer and Hughes, 2010) which were made more quickly and required fewer cognitive resources (Beer and Hughes, 2010). Weaker self-serving evaluations had been argued to be related to the involvement of more cognitive resources in judgments (Beer and Hughes, 2010). Reduced self-serving evaluations in recipient role in Kasof and Lee (1993) suggested that more complicated self-evaluation processes associated with more cognitive resources relative to heuristic judgments were engaged in the judgments.

The present study aimed to explore the neural mechanism underlying how the actor-recipient role would affect self-processing in emotional situations using functional MRI. Participants were scanned while they were presented with positive and negative interpersonal events and were asked to rate “how likely it is that the actor/the recipient is that kind of person who caused the interpersonal event”. In half of the trials, “self” was randomly assigned to an actor or recipient (e.g., “I hit Mary” or “Lisa hits me”) and “self” was the evaluation target. In the other half of the trials, other-relevant events were presented (e.g., “Ted hits Paul”) and “other” was the evaluation target. We predicted greater self-serving evaluations for actor than for recipient and this effect was stronger for negative relative to positive information based on the abundant evidence for negativity bias (Carretié et al., 2001; Hilgard et al., 2014). At the neural level, we expected that activities in mPFC, ACC and OFC would be associated with the impact of the actor-recipient role on self-processing in emotional interpersonal events, especially in negative events. Previous studies have claimed that dmPFC was a region associated with self-evaluation processes (Northoff and Bermpohl, 2004; Korn et al., 2012) and it has also been argued that people may consume more cognitive resources to engage in self-evaluation when they played the role of recipient. Therefore, the self-related activations (self-other) in dmPFC would be greater when the role of recipient relative to actor was played in negative interpersonal events and greater self-related dmPFC activations would be associated with longer reaction time (RT) for self-related evaluation processes. In addition, we expected that activities in OFC would be greater when participants made less self-serving judgments in the recipient relative to actor role, based on previous findings that OFC was associated with accurate self-view (Beer et al., 2006; Beer, 2007; Beer and Hughes, 2010).

## Material and Methods

### Participants

Twenty-nine right-handed volunteers from the university community with normal or corrected-to-normal vision participated in this experiment. Four participants had to be excluded due to excessive head movements, leaving 25 subjects for analyses (13 females, aged from 20 to 30 years, *M* = 24.16, *SD* = 2.59). The twenty-five participants included in the data analysis consisted of 5 undergraduate students (all females) and 20 graduate students (12 males and 8 females). None of the participants reported a significant abnormal neurological history. All the participants gave informed consent before scanning and were paid RMB50 for their participation. This study was approved by the Ethical Committee of East China Normal University.

### Materials

Verbs with mean valence ratings of larger than 6 (positively) and smaller than 3 (negatively) based on 9-point pleasant-unpleasant ratings were selected from Chinese Affective Words System (CAWS; Wang et al., 2008). Forty two-character Chinese verbs (20 positively valenced and 20 negatively valenced) were used in the present study. These two sets of verbs differed in valence (*t* = 75.22, *p* < 0.001) but equated for arousal, familiarity and frequency.

These verbs were used to construct one-sentence interpersonal events. Each sentence was comprised of one subject, one verb and one object, such as “Ted hits Paul”. Each verb was used four times to construct four different types of conditions, according to the crossing of all possible combinations of evaluation Target (Self vs. Other) and Role (Actor vs. Recipient). An example of a verb with positive valence (i.e., like) is given below with four respective sentences and their judgment questions.

A1. **I like John**. How likely is it that **I** am that kind of person?
A2. **Tom likes me**. How likely is it that **I** am that kind of person?
B1. **Ted likes Paul**. How likely is it that **Ted** is that kind of person?
B2. **Ted likes Paul**. How likely is it that **Paul** is that kind of person?

For self-relevant events, “self” was randomly assigned as the actor (e.g., A1) or recipient (e.g., A2) and self was the target of evaluation. For other-relevant events, both the actor (e.g., B1) and the recipient (e.g., B2) would become the targets of evaluation separately. Similarly, the negatively valenced verbs were also used to construct four different types of conditions.

### Procedure

**Figure 1:**
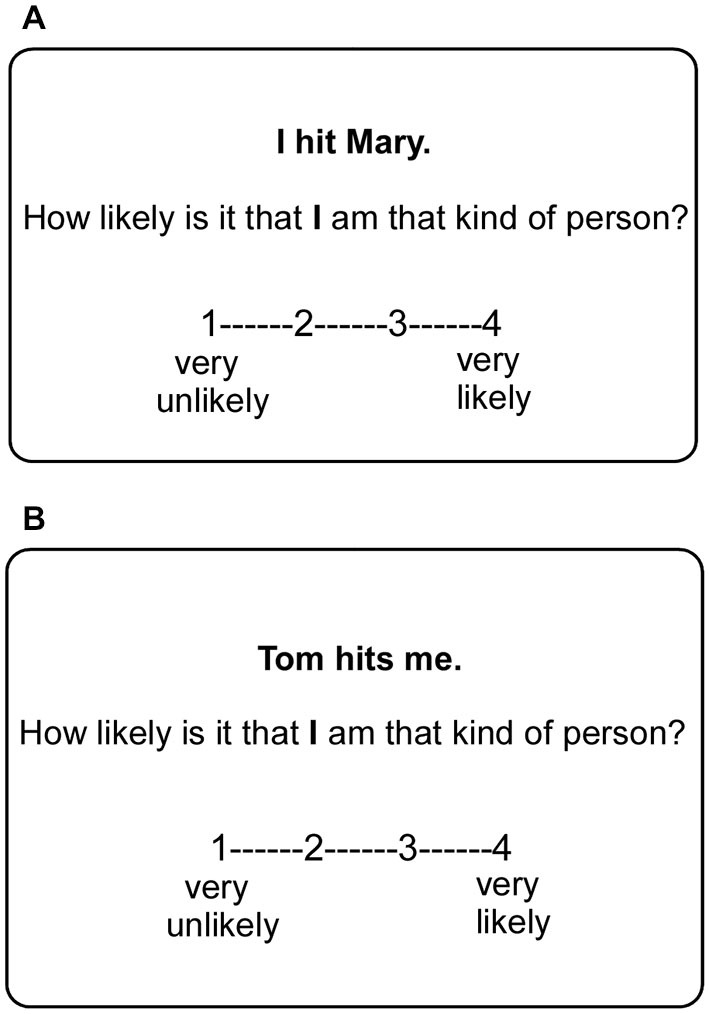
**Examples of situation stimuli used in the experiment**. Each stimulus included a one-sentence interpersonal event, a question and a 4-point scale. **(A)** Self-relevant negative interpersonal event and the evaluation target “self” played the role of actor. **(B)** Self-relevant negative event and the evaluation target “self” played the role of recipient.

Participants were asked to complete 160 trials in the scanner with 20 trials in each condition. In each trial, participants were presented with a one-sentence interpersonal event, a question and a 4-point scale (Figure 1). They were required to read the sentence and to answer the question within 5 s on the 4-point scale by pressing the corresponding button (1 = very unlikely, 2 = moderately unlikely, 3 = moderately likely, 4 = very likely). Once the participant responded, a red circle would appear around the selected number and lasted for 1.5 s. Each trial was jittered with inter-stimulus intervals from 2 s to 6 s, during which a black fixation cross was presented against a white background. All the trials were presented in a random order.

### fMRI Image Acquisition and Analysis

Scanning was carried out on a 3T Siemens scanner at the Functional MRI Lab (East China Normal University, Shanghai). For functional images, 35 slices were acquired using a gradient-echo echo-planar imaging (EPI) sequence (TR = 2200 ms, TE = 30 ms, FOV = 220 mm, matrix size = 64 × 64, slice thickness = 3 mm, gap = 0.3 mm). Before the functional run, a high-resolution structural image was acquired using a T1-weighted, multiplanar reconstruction (MPR) sequence (TR = 1900 ms, TE = 3.42 ms, 192 slices, slice thickness = 1 mm, FOV = 256 mm, matrix size = 256 × 256).

Data preprocessing and statistical analyses were performed with Statistical Parametric Mapping (SPM12, Wellcome Department of Cognitive Neurology, London). Preprocessing included discarding the first five functional images to allow for scanner equilibrium effects, rigid-body motion correction, spatial normalization into the MNI space (resampled at 2 × 2 × 2 mm^3^ voxels), and spatial smoothing (using an 8-mm full-width half maximum isotropic Gaussian kernel). A high-pass filter with a cutoff period of 128 s was applied.

At the first level, eight conditions were defined according to Target (Self vs. Other), Role (Actor vs. Recipient) and Valence (Positive vs. Negative). They were modeled using a canonical hemodynamic response function with a temporal derivative. We have chosen the onset of the stimulus as the onset time point and the RT from the stimulus onset to button press as the duration (epoch with variable time length). Six regressors modeling movement-related variance and one modeling the overall mean were also employed in the design matrix. A general linear model analysis created 8 contrast images for each participant summarizing differences of interest. The eight first-level contrast images from each participant were then analyzed at the second level employing a random-effects model (flexible factorial design in SPM12).

We used *F*-contrast to analyze the Target × Valence interaction effect across roles. Then, in order to examine the effect of the actor-recipient role on self-processing in emotional situations, *F*-contrast was further used to examine the Target × Valence × Role interaction. Areas of activation were identified as significant only if they passed the threshold of *p* < 0.05 family-wise error (FWE) corrected for multiple comparisons at the cluster level with an underlying voxel level of *p* < 0.0001 uncorrected, unless otherwise indicated. Marsbar toolbox (Brett et al., 2002) was used to extract beta-values from the activated brain regions.

## Results

### Task Performance

A 2(Target: Self vs. Other) × 2(Valence: Positive vs. Negative) × 2(Role: Actor vs. Recipient) repeated measures ANOVA revealed that participants’ attribution ratings were characterized by a significant Target × Valence × Role interaction (*F*_(1,24)_ = 31.79, *p* < 0.001) (Table 1). When the evaluation target took the role of actor, repeated measures ANOVA revealed that the interaction between target and valence was significant (*F*_(1,24)_ = 103.01, *p* < 0.001). Paired-sample *t* test revealed that participants’ attribution ratings of self were significantly higher than those of other in positive events (*t*_(24)_ = 2.68, *p =* 0.01), and participants’ attribution ratings were significant lower to self than other in negative events (*t*_(24)_ = 9.68, *p* < 0.001). When the evaluation target played the role of recipient, repeated measures ANOVA revealed that the interaction between target and valence was also significant (*F*_(1,24)_ = 42.84, *p* < 0.001). Paired-sample *t* test revealed that participants’ attribution ratings were significantly lower to self than other in negative events (*t*_(24)_ = 7.68, *p* < 0.001), whereas there was no significant difference between self and other in positive events (*t*_(24)_ = 0.83, *p* = 0.42).

**Table 1:** **Means and standard deviations of participants’ attribution ratings of self and other when the evaluation target played the role of actor and recipient in positive and negative interpersonal events**.

	Positive		Negative	
	Self	Other	Self	Other
Actor	3.27 ± 0.44	3.07 ± 0.32	1.63 ± 0.35	2.77 ± 0.50
Recipient	3.11 ± 0.36	3.06 ± 0.29	1.76 ± 0.40	2.52 ± 0.36

Moreover, paired-sample *t* test revealed a greater attribution rating difference (self-other) when participants played the role of actor relative to recipient in positive (*t*_(24)_ = 3.06, *p* = 0.005) and negative (*t*_(24)_ = 4.85, *p* < 0.001) interpersonal events. These results indicated that participants were more likely to endorse positive events to self and isolate self from negative events, and this attribution bias was greater when participants played the role of actor relative to recipient.

Repeated measures ANOVA revealed that participants’ RT was characterized by main effects of Target (*F*_(1,24)_ = 26.93, *p* < 0.001), Valence (*F*_(1,24)_ = 10.05, *p* = 0.004) and Role (*F*_(1,24)_ = 36.11, *p* < 0.001) (Table 2). Judgments in the self condition (*M* = 2319 ms, *SD* = 93) were made significantly faster than those in the other condition (*M* = 2716 ms, *SD* = 122). Judgments in the positive condition (*M* = 2475 ms, *SD* = 104) were made significantly faster than those in the negative condition (*M* = 2560 ms, *SD* = 100). Judgments in the actor condition (*M* = 2439 ms, *SD* = 98) were also made significantly faster than those in the recipient condition (*M* = 2596 ms, *SD* = 106).

**Table 2:** **Means and standard deviations of participants’ RT (ms) of judgment for self and other when the evaluation target played the role of actor and recipient in positive and negative interpersonalevents**.

	Positive		Negative	
	Self	Other	Self	Other
Actor	2213 ± 98	2613 ± 122	2246 ± 93	2684 ± 121
Recipient	2332 ± 102	2742 ± 129	2485 ± 101	2827 ± 127

### fMRI Results

#### Target × Valence Interaction

**Figure 2:**
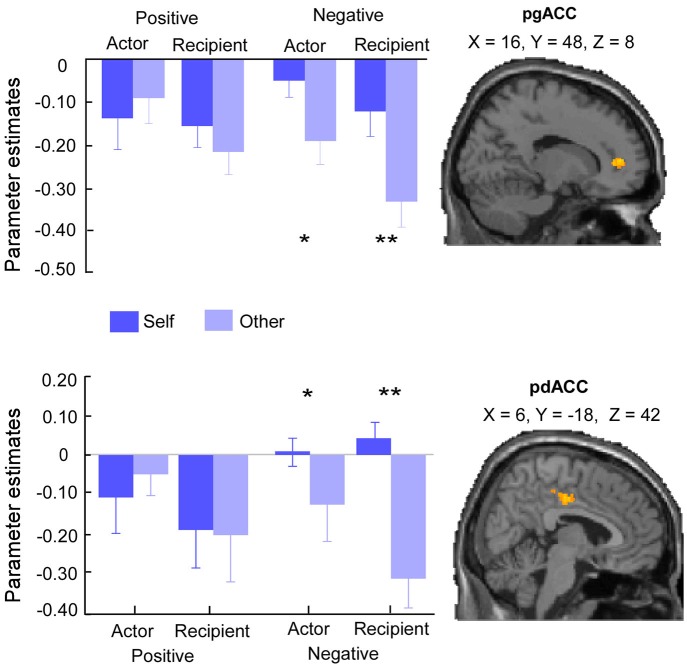
**Brain activities in Target × Valence interaction effect across roles**. Activities in pgACC (MNI 16, 48, 8) and pdACC (MNI 6, −18, 42) were greater for self than other only when interpersonal events were negative. Error bars indicated standard errors of mean beta-values. Asterisks indicated that beta-values were greater for self than other(*p < 0.05, **p < 0.001).

The *F*-contrast of Target (Self vs. Other) × Valence (Positive vs. Negative) interaction across roles revealed significant activations in pregenual ACC (pgACC) (MNI 16, 48, 8) and posterior dACC (pdACC) (MNI 6, −18, 42) (Figure 2; Table 3). Additionally, this contrast revealed significant activations in postcentral gyrus (MNI 44, −24, 54), lingual gyrus (MNI −12, −70, 4), thalamus (MNI 12, −18, 16) and middle temporal gyrus (mTG) (MNI 50, −32, −2).

**Table 3:** **Identification of BOLD signal changes association with the Target × Valence interaction and Target × Valence × Role interaction**.

	Peak Activation		
Brain Region	*X*	*Y*	*Z*	*F*-Value	Voxels
**Target × Valence interaction**
R Postcentral Gyrus	44	−24	54	57.69	3534
L Lingual Gyrus	−12	−70	4	50.51	1898
R Pregenual Anterior Cingulate Cortex	16	48	8	34.92	731
R Posterior Dorsal Anterior Cingulate Cortex	6	−18	42	34.20	650
R Thalamus	12	−18	16	33.68	245
R Middle Temporal Gyrus	50	−32	−2	30.60	132
**Target × Valence × Role interaction**
R Lingual Gyrus	16	−58	−2	26.15	173
L Dorsal Medial Prefrontal Cortex	−18	38	50	23.09	80
R Calcarine Gyrus	16	−68	8	20.92	54
R Orbital Frontal Cortex	−32	60	0	20.90	69

*Note. Coordinates (mm) are in MNI space. L = left hemisphere; R = right hemisphere. All reported clusters are cluster-level family wise error (FWE) corrected for multiple comparisons at *p* < 0.05*.

Parameter estimates across pgACC and pdACC were extracted. Paired-sample *t* test revealed similar pattern of activations in pgACC and pdACC, that is, in both the actor and recipient conditions, the activities in these two regions were greater for self than other only in negative events (actor:* t*_(24)_ = 3.41, *p* = 0.002, and recipient: *t*_(24)_ = 4.95, *p* < 0.001 for pgACC; actor:* t*_(24)_ = 2.47, *p* = 0.02, and recipient: *t*_(24)_ = 5.16, *p* < 0.001 for pdACC) but not in positive events (actor: *t*_(24)_ = 1.04, *p* = 0.31, and recipient: *t*_(24)_ = 1.76, *p* = 0.10 for pgACC; actor: *t*_(24)_ = 0.36, *p* = 0.72, and recipient: *t*_(24)_ = 1.23, *p* = 0.23 for pdACC).

#### Target × Valence × Role Interaction

**Figure 3:**
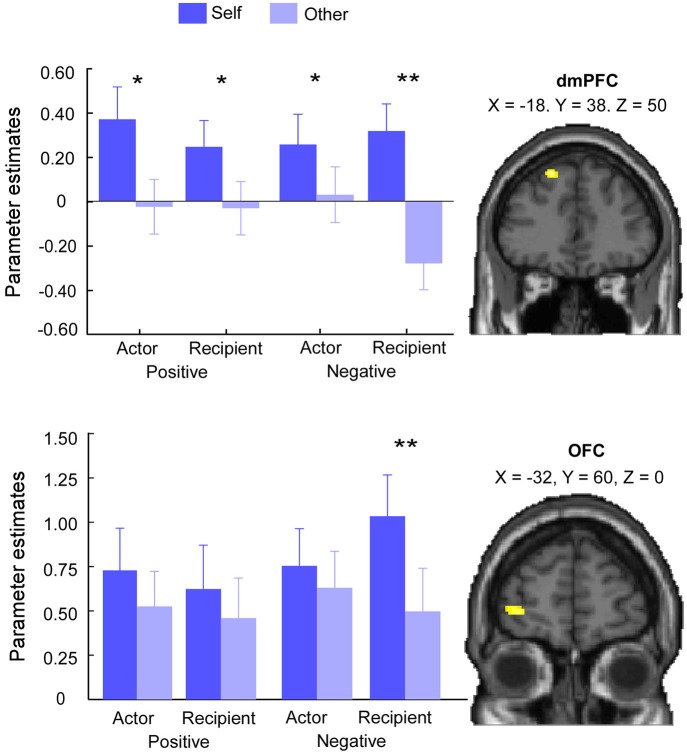
**Brain activities in Target × Valence × Role interaction**. Activities in dmPFC (MNI −18, 38, 50) were greater for self than other when the actor or recipient role was played in both positive and negative events. Activities in orbitofrontal cortex (OFC) (MNI −32, 60, 0) were greater for self than other only when the evaluation target played the role of recipient in negative interpersonal events. Error bars indicated standard errors of mean beta-values. Asterisks indicated that beta-values were greater for self than other (*p < 0.05, **p < 0.001).

**Figure 4:**
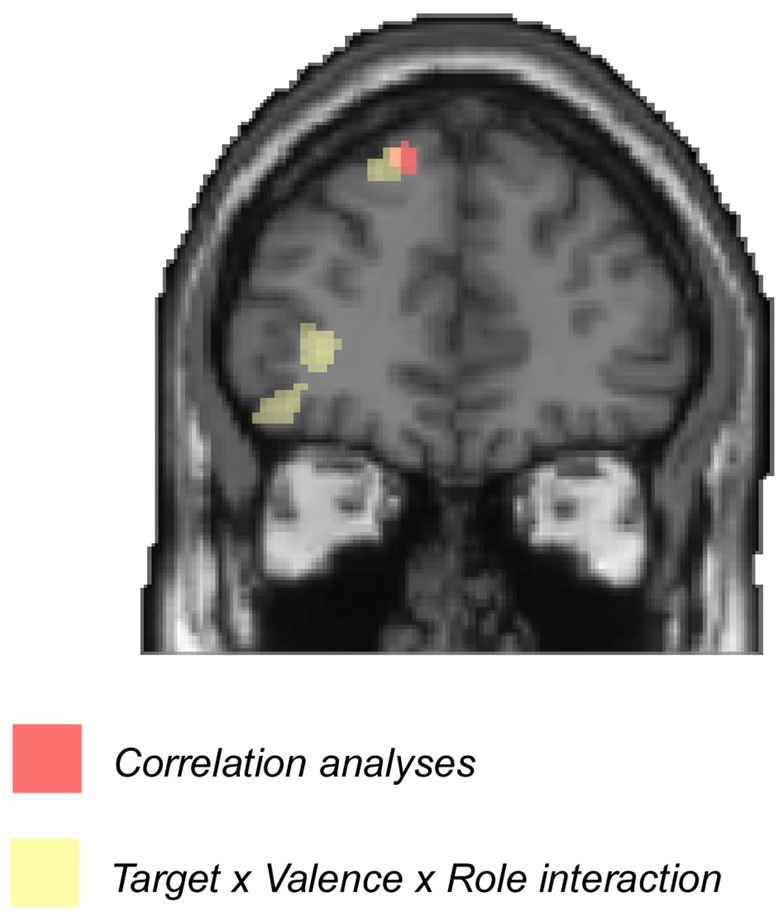
**Overlap**. Clusters in dmPFC overlapped between correlation analyses and the Target × Valence × Role interaction (*p* < 0.001, uncorrected).

The *F*-contrast of Target × Valence × Role interaction revealed significant activations in dmPFC (MNI −18, 38, 50) and OFC (MNI −32, 60, 0) (Figure 3; Table 3). Additionally, this contrast revealed activations in lingual gyrus (MNI 16, −58, −2) and calcarine gyrus (MNI 16, −68, 8). We extracted the parameter estimates across dmPFC and OFC. Paired-sample *t* test revealed that, in both positive and negative events, the brain activity in dmPFC was greater for self than other in both the actor (positive: *t*_(24)_ = 3.74, *p* = 0.001; negative: *t*_(24)_ = 2.08, *p* = 0.05) and recipient conditions (positive: *t*_(24)_ = 3.57, *p* = 0.002; negative: *t*_(24)_ = 6.93, *p* < 0.001). Interestingly, paired-sample *t*-test revealed greater self-related dmPFC activations (self-other) for the recipient relative to actor in negative interpersonal events (*t*_(24)_ = 4.61, *p* < 0.001) but not in positive events (*t*_(24)_ = 1.77, *p* = 0.09). The dmPFC has been argued to be associated with self-evaluation processes (Northoff and Bermpohl, 2004; Beer and Hughes, 2010; Han et al., 2010). It could be assumed that people consumed more cognitive resources to engage in self-evaluation processes when they played the role of recipient in negative events. Correlation analyses were performed to determine brain regions whose BOLD signal change detected from the self *vs*. other contrast when the recipient role was played in negative events varied with the corresponding RT (after log-transformation). A significant cluster of activation was detected in dmPFC (MNI −12, 44, 48, 22 voxels, survived a voxel-level intensity threshold of *p* < 0.001, uncorrected), indicating greater self-related dmPFC activations were associated with longer RT for self relative to other for the recipient in negative events. We then overlapped activations from correlation analyses with activations from the *F*-contrast of Target × Valence × Role interaction. Clusters in dmPFC overlapped (Figure 4).

Paired-sample *t* test revealed that, in positive events, the brain activity in OFC showed no difference for self than other in both the actor (*t*_(24)_ = 1.89, *p* = 0.07) and recipient (*t*_(24)_ = 1.38, *p* = 0.18) conditions. In negative events, the brain activity in OFC was greater for self than other only in the recipient condition (*t*_(24)_ = 5.98, *p* < 0.001) but not in the actor condition (*t*_(24)_ = 0.65, *p* = 0.52). Moreover, paired-sample *t* test revealed that OFC showed greater self-related activations when participants played the role of recipient relative to actor in negative interpersonal events (*t*_(24)_ = 3.69, *p* = 0.001) but not in positive events (*t*_(24)_ = 0.84, *p* = 0.41).

## Discussion

The present study explored the impact of the actor-recipient role on neural responses to self in emotional situations. Results of attribution ratings revealed that people tended to endorse positive events as self-relevant only when they performed the role of actor, whereas they were more likely to isolate self from negative events when they played the role of actor relative to recipient, indicating greater self-serving evaluations when actor role was played in negative situations. Activities in pgACC and pdACC were greater for self than other only when the interpersonal events were negative. Moreover, activities in dmPFC were greater for self than other when the actor or recipient role was played in both positive and negative events, whereas activities in OFC were greater while responding to self than to other only when the evaluation target played the role of recipient in negative interpersonal events. Importantly, OFC and dmPFC showed greater self-related activations when participants played the role of recipient relative to actor in negative interpersonal events. These results showed that the actor-recipient role affected neural responses to self in emotional situations, especially when a recipient role was played in negative situations.

Previous research had claimed that people had the tendency to make claims that cast the self in a favorable light (Sedikides et al., 1998; Heine et al., 1999; Duval and Silvia, 2002; Mezulis et al., 2004). People have a need to view themselves positively (Heine et al., 1999; Mezulis et al., 2004). However, participants’ positive self-image would be threatened when they are involved in negative interpersonal events. Thus, encountering negative events would conflict with the need to maintain individuals’ positive outlook. In order to diminish the conflict, people tend to isolate themselves from negative events (Miller and Ross, 1975; Cunningham et al., 1979; Duval and Silvia, 2002; Mezulis et al., 2004). This tendency was confirmed by attribution ratings data. That is, participants were more likely to endorse negative events as self-irrelevant. At the neural level, greater activations of pgACC and pdACC were found during response to self than to other only when the interpersonal events were negative. ACC has long been conceived in neuroimaging studies to have a regulatory role with respect to limbic regions. It is involved in generating negative emotional responses and is important for emotional conflict monitoring and regulation (Carter et al., 1998; Bush et al., 2000; Northoff and Bermpohl, 2004; Etkin et al., 2011). The greater self-related activations in pgACC and pdACC in negative events further confirmed the role of ACC in monitoring and regulating the conflict between negative information and the maintenance of a positive outlook.

It has been suggested that the dmPFC is engaged in self-evaluation and self-related reappraisal (Northoff and Bermpohl, 2004; Northoff et al., 2006; Han et al., 2010; Korn et al., 2012). The more efforts are involved in the cognitive processing in self-evaluation, the greater dmPFC is activated (Beer and Hughes, 2010). In the current study, dmPFC exhibited greater self-related activations in the recipient relative to actor condition only in negative interpersonal events. Correlation analysis further revealed increased self-related activations in this dmPFC for people with longer RT for self relative to other for the recipient in negative events (observed at a looser threshold), indicating greater cognitive self-evaluation processing in negative events when the recipient role was played. Moreover, there were reduced self-serving evaluations for the recipient relative to actor in negative events found in our study. Previous studies had demonstrated that self-serving evaluations were heuristic judgments and self-serving judgments required fewer cognitive resources than the other judgments (Dunning et al., 1989; Chambers and Windschitl, 2004; Beer and Hughes, 2010). Reduced self-serving evaluations for the recipient relative to actor in negative events in our study indicated more cognitive processing when self received negative events, providing further support for the role of dmPFC in self-evaluation.

The current study also showed that activities in OFC were greater while responding to self than to other only when the evaluation target played the role of recipient in negative interpersonal events. That is, OFC was modulated by the actor-recipient role in negative interpersonal events. Previous studies had shown that OFC was associated with accurate self-view (Beer et al., 2006; Beer, 2007; Beer and Hughes, 2010). More heuristic self-serving evaluations were associated with less activities in OFC (De Martino et al., 2006; Beer and Hughes, 2010). In the present research, participants made more heuristic self-serving judgments when they played the role of actor than when they played the role of recipient in negative interpersonal events. This results were consistent with the neural activations in OFC, that is, the self-related activations were greater when the role of recipient was played than that of actor was played in negative interpersonal events.

Previous studies had revealed the engagement of mTG in attribution decisions (Blackwood et al., 2003; Harris et al., 2005; Seidel et al., 2010; Cabanis et al., 2013) and activities in thalamus in self-related and/or emotion-related processing (Seidel et al., 2010; Somerville et al., 2010; Turk et al., 2011; Korn et al., 2012). These brain regions were also activated in the interaction between target and valence in our data, providing further support for the role of mTG and thalamus in self-related emotional attribution. In addition, this study has a limitation which should be noted. Arousal-matched positive and negative materials were used in the present research. However, valence and arousal as two dimensions of emotion have been argued to work in an interactive way (Robinson et al., 2004; Citron et al., 2014). Future studies should further examine the impact of the actor-recipient role on self-related processing in different valence and arousal situations.

In conclusion, the current study further illustrated neural models of social cognition by examining the impact of the actor-recipient role on self-processing in emotional situations. The pgACC, pdACC, OFC, and dmPFC were involved in emotional self-processing. Self-related pgACC and pdACC activations in negative events indicated its association with monitoring conflict in negative self-related processing. More importantly, dmPFC and OFC were modulated differently by the actor-recipient role in emotional self-processing, with dmPFC associated with self-evaluation processes and OFC engaged in accurate self-view. Taken together, these results demonstrated that the actor-recipient role affected neural responses to self in emotional situations.

## Author Contributions

All authors were involved in the design and implementation of the study and the writing of the manuscript. Authors Xiaoyan Wang, Li Zheng, Xuemei Cheng, Lin Li and Xiuyan Guo devised the concept and supervised the study. Authors Xuemei Cheng and Qianfeng Wang collected the imaging data. Authors Xiaoyan Wang, Li Zheng carried out the analysis. Authors Li Zheng, Xuemei Cheng, Lin Li and Xiuyan Guo joined in the interpretation of data. Authors Lining Sun joined in the writing of the manuscript.

## Conflict of Interest Statement

The authors declare that the research was conducted in the absence of any commercial or financial relationships that could be construed as a potential conflict of interest.
